# Individual and temporal variability of the retina after chronic bilateral common carotid artery occlusion (BCCAO)

**DOI:** 10.1371/journal.pone.0193961

**Published:** 2018-03-16

**Authors:** Sergio Crespo-Garcia, Nadine Reichhart, Sergej Skosyrski, Marco Foddis, Jim Wu, Aleksandar Figura, Christina Herrspiegel, Martina Füchtemeier, Celeste Sassi, Ulrich Dirnagl, Antonia M. Joussen, Olaf Strauss

**Affiliations:** 1 Department of Ophthalmology, Experimental Ophthalmology, Charité Universitätsmedizin Berlin, Berlin, Germany; 2 Department of Neurology, Experimental Neurology, Charité Universitätsmedizin Berlin, Berlin, Germany; University of Florida, UNITED STATES

## Abstract

Animal models of disease are an indispensable element in our quest to understand pathophysiology and develop novel therapies. *Ex vivo* studies have severe limitations, in particular their inability to study individual disease progression over time. In this respect, non-invasive *in vivo* technologies offer multiple advantages. We here used bilateral common carotid artery occlusion (BCCAO) in mice, an established model for ischemic retinopathy, and performed a multimodal *in vivo* and *ex vivo* follow-up. We used scanning laser ophthalmoscopy (SLO), ocular coherence tomography (OCT) and electroretinography (ERG) over 6 weeks followed by *ex vivo* analyses. BCCAO leads to vascular remodeling with thickening of veins starting at 4 weeks, loss of photoreceptor synapses with concomitant reduced b-waves in the ERG and thinning of the retina. Mononuclear phagocytes showed fluctuation of activity over time. There was large inter-individual variation in the severity of neuronal degeneration and cellular inflammatory responses. *Ex vivo* analysis confirmed these variable features of vascular remodeling, neurodegeneration and inflammation. In summary, we conclude that multimodal follow-up and subgroup analysis of retinal changes in BCCAO further calls into question the use of *ex vivo* studies with distinct single end-points. We propose that our approach can foster the understanding of retinal disease as well as the clinical translation of emerging therapeutic strategies.

## Introduction

Although there is a rapid and exponential gain in knowledge of basic science, the translation of experimental results to clinic is still challenging and often fails. For many years, basic animal research was based on fixed or limited end-point read-outs in disease models although there is an urgent need for exploratory studies to understand disease progression. Research aiming to understand the individual time-course at disease onset, even in animal models, would provide a very useful insight to research with a high value for clinical translation.

*In vivo* monitoring and evaluation of pathological parameters are driving recent advances in animal-based translational research. These parameters are promising biomarkers as they are often present before the onset of the disease. In research, the retina is a privileged organ because of its easy optical and electrophysiological accessibility. The retina permits non-invasive, multimodal, and almost simultaneous, analysis of neuronal activity, blood vessel integrity and micro-structure [1–3] by means of electroretinography (ERG), optical coherence tomography (OCT) and laser-scanning ophthalmoscopy (SLO). These methods are routinely used for diagnostic and prognostic purposes in the clinic. Thus, their use in animal models permits a correlation of clinically relevant parameters and molecular pathology. Furthermore, these methods of *in vivo* analysis allow follow-up studies employing the same animal and thus might have more of a translational impact.

The benefits of *in vivo* investigation of the retina are not only limited to the understanding of ophthalmic disease. Retinal function assessment has become increasingly important in the investigation of other end-organs such as kidney, heart or brain due to the correlation between changes in the retina and the development of systemic diseases. New methods to monitor structure and perfusion of the retina currently employed in the clinic are now also available for animal models. Thus, evaluation of the potential of multimodal retina analysis is a prerequisite to fully exploit the possibilities of *in vivo* assessments in translational research.

We used a mouse model of ischemic retinopathy. Chronic hypoxia leads to retinal damage and it is a common end-point in multiple systemic and local pathologies [4]. One of the reasons for hypoxia in the retina is blood flow reduction associated to diseases such as carotid artery disease. This ischemic condition leads to visual impairment. The mouse model selected has a bilateral common carotid artery occlusion (BCCAO) to mimic chronic hypoperfusion of the retina and the brain. This model has been traditionally used to understand neurodegeneration in a context of ischemic retinopathy as well as of brain hypoperfusion [5–10]. The focus of our study is the *in vivo* follow-up in the retina of the most common neurodegenerative, vascular and inflammatory features during a time-course of 6 weeks after occlusion. We aim to discover new features of disease progression, and to combine our findings with observations from other groups. The multimodal frequent analysis approach will help create a complete picture of the pathology and to gain insight to patho-mechanisms in BCCAO and in other translational animal models with varying degrees of impact.

## Methods

### Animal model and experimental design

All experiments were designed and performed in compliance with the ARVO Statement for the Use of Animals in Ophthalmic and Vision Research, and approved by the Landesamt für Gesundheit und Soziales (LaGeSo, G 0068/12) local authorities. All experiments were in compliance with the ARRIVE guidelines (S1 File).

Male mice underwent coil implantation at the age of 10 weeks, and were housed in 12-hour light/dark cycle facilities with free access to water and chow. After surgery, animals were regularly checked and a protocol for humane endpoint of the experimental subjects was established (S2 File). The strains employed in the study were C57/Bl6J (Charles River Erkrath, Germany)) or MacGreen [11] (B6N.Cg-Tg(*Csf1r*-EGFP)1Hume/J; bred in house) with common genetic background. Transgenic MacGreen mice were employed in *in vivo* studies. This strain harbors an enhanced fluorescent green protein (EGFP) reporter at the site of the *Csf1r* promoter, which is expressed in myeloid-derived macrophages. Both strains were tested negative for RD8 mutation [12].

Animals were evaluated weekly *in vivo* prior to surgery and after until week 6. *Ex vivo* read-outs were performed at week 1 and week 6 after surgery.

### Chronic bilateral common carotid artery occlusion

Carotid stenosis was induced wrapping both carotid arteries with micro-coils as described by Füchtemeier et al. as a model for chronic bilateral common carotid artery occlusion (BCCAO) and cerebral hypoperfusion [6]. In brief, animals were anesthetized and two small incisions in the neck allowed the implantation of the micro-coils (N = 42; 180 μm inner diameter, Sawane Spring Company, Hamamatsu, Japan) on both carotid arteries. Sham animals (N = 28) underwent the same surgical procedure without micro-coil implantation and served as control.

### Scanning laser ophthalmoscopy (SLO) and optical coherence tomography (OCT)

Pupils were dilated using 2.5% phenylephrin-hydrochlorid and 0.5% tropicamid (Charité Apotheke, Berlin, Germany). Anesthesia and subsequent *in vivo* imaging was performed using a scanning laser ophthalmoscope Spectralis HRA-OCT (Heidelberg Engineering, Heidelberg, Germany) as described previously by our group [13]. In short, anesthetized animals were located at a customized platform attached to the ophthalmoscope with a 30° lens. Retina was investigated: 1) with AF mode (488 nm) before (auto-fluorescence, AF) and after fluorescein injection (Alcon, Berlin, Germany) for fundus angiography (FAG) or 2) with the mode for optical coherence tomography (OCT) for retinal thickness studies. All images and retinal thickness mapping were digitalized using the Heidelberg Eye Explorer software (Heidelberg Engineering).

### Vein-artery ratio analysis

In the angiographs, veins and arteries were identified phenotypically [14]. Vein-artery ratios were calculated in each individual fundus angiography (26 eyes with BCCAO and 12 control eyes, 1 image per time-point per eye) in masked fashion by an independent investigator. Measurements were performed using ImageJ (NIH, Bethesda, Maryland, US) setting two concentric reference circles. The first circle was centered at the optic nerve head (ONH), and the second circle served to measure the vessel width between the intersection points. The ratio was calculated from mean (AU) values of the total artery and vein diameter from each photography.

The increase in vein/artery ratio over time was verified *ex vivo* in flat-mount preparations at the end point of the experiment (4 affected coil and 3 sham animals).

Eyes were enucleated and fixed in PFA 4% during 15 min. Cornea and lens were gently removed and discarded. The posterior part of the eye was flattened by doing four radial cuts and the neural retina was separated from the choroid sectioning the optic nerve. Retina was blocked using normal goat serum (1 h, RT) and then isolectin-B_4_ antibody (see Table 1) was applied overnight at 4°C. After repeated washes, retina was mounted in a flat position onto a glass-slide and visualized using a Zeiss Axio Imager (Zeiss, Jena, Germany) and quantified as the in vivo images.

**Table 1:** Primary antibodies.

Antibody against	Dilution	Unmasking treatment	Supplier	Cat #
Mouse anti-CALB	1:500	Proteinase K	Sigma	C9848
Rabbit anti-Caspase 3	1:250	Citrate buffer (pH 6)	Cell Signalling	9662
Rabbit anti-Cleaved Caspase 3	1:250	Citrate buffer (pH 6)	Cell Signalling	9661
Rabbit anti-CTBP2	1:500	Proteinase K	Abcam	ab128871
Goat anti-GFP	1:200	None	Abcam	ab6662
GS Isolectin-B_4_	1:200	None	Invitrogen	l21411
Mouse anti-GFP	1:200	None	Abcam	ab291
Rat anti-CD11b	1:200	None	Antibodies online	ABIN474860
Rat anti-PlGF	1:100	Proteinase K	Abcam	ab51654
Rabbit anti-Iba1	1:500	EDTA buffer (pH 9)	Wako	01–1974
Rabbit anti-PKCα	1:100	Citrate buffer (pH 6)	Santa Cruz	sc-208
Rabbit anti-VEGF-A	1:100	Citrate buffer (pH 6)	Booster Immuno	PA1080
Rabbit anti-VGLUT1	1:250	Citrate buffer (pH 6)	Abcam	ab104898

### Quantitative PCR

Eyes were enucleated at the end-point of the experiment (6 weeks) and immediately frozen at -80°C. Retina RNA isolation (RNEasy Mini Kit; Qiagen, Hilden, Germany) and cDNA synthesis (QuantiTect Reverse Transcription Kit, Qiagen) were carried out according to manufacturer’s instructions. Quantitative real time PCR (qPCR) was done using QuantiTect Sybr Green PCR Master Mix (Qiagen) on a Rotor Gene Q (Qiagen). All reactions were prepared in triplicate. The ddCT method was applied to calibrate the target genes and target gene expression was normalized to GAPDH [15]. Primers are listed in Table 2. For gene expression analysis, only BCCAO animals characterized as affected by means of *in vivo* data were considered for analysis.

**Table 2:** Primer sequences.

Gene	Sequence	
*Ccl2*	F	TCACCTGCTGCTACTCATTCA
	R	CACTGTCACACTGGTCACTCC
*Cd68*	F	AGGGTGGAAGAAAGGCTTGG
	R	ACTCGGGCTCTGATGTAGGT
*Cd86*	F	CAGCACGGACTTGAACAACC
	R	CTCCACGGAAACAGCATCTGA
*Flt1*	F	AGGGTGTCTATAGGTGCCGA
	R	ACTTCGGAAGAAGACCGCTT
*Gapdh*	F	AACTTTGTGAAGCTCATTTCCTGGTAT
	R	CCTTGCTGGGCTGGGTGGT
*Il1b*	F	GACCTTCCAGGATGAGGACA
	R	AGGCCACAGGTATTTTGTCG
*Il4r*	F	GAGGGACCTGGCTTCTGATT
	R	CCTTGATGCTCCCAGATCCA
*Il6*	F	CAGAGGATACCACTCCCAACA
	R	CCAGTTTGGTAGCATCCATC
*Plgf*	F	AGATCTTGAAGATTCCCCCCA
	R	TTCCCCTTGGTTTTCCTCCTT
*sFlt1*	F	GAAGACTCGGGCACCTATGC
	R	CCGCAGTGCTCACCTCTAAC
*Tnfa*	F	CGCGACGTGGAACTGGCAGAA
	R	GTGGTTTGCTACGACGTGGGCT
*Vegfa*	F	CAGCTATTGCCGTCCGATTGAGA
	R	TGCTGGCTTTGGTGAGGTTTGAT
*Vegfb*	F	GCCAGACAGGGTTGCCATAC
	R	GGAGTGGGATGGATGATGTCAG

### Immunohistochemistry

Eyes were enucleated, fixed in PFA 4% overnight and embedded in paraffin. Sections of 5 μm thickness were deparaffinized and stained using standard procedure for H/E or with different antibodies. Prior to immunodetection, sections were treated with different antigen retrieval protocols (see Table 1) and blocked with BSA 5% (1 h, RT). Primary antibodies were applied overnight and detected with species-appropriate fluorescence-conjugated secondary antibodies (1 h, RT; see Table 3). The different antibodies and their dilutions are indicated in Tables 1 and 3. Isotype controls are shown in the S1 Fig. Images were digitalized using a Zeiss Axio Imager (Zeiss, Jena, Germany) microscope with Apotome, and processed with Zen-Lite 2012 software (Zeiss).

**Table 3:** Secondary antibodies.

Antibody against	Dilution	Supplier	Cat #
Donkey anti-Rabbit AF488	1:10000	Invitrogen	A-21206
Goat anti-mouse AF546	1:5000	Invitrogen	A-11030

### Evaluation of the outer plexiform layer loss

Loss of the outer plexiform layer (OPL) was assessed on sections stained against H/E. 5–10 representative sagittal sections per eye were scored to determine the degree of OPL-loss in the retina. Grade I stands for no structural alterations detected, Grade II has focal alterations in the OPL (loss of 0–25% of the OPL), Grade III is defined by loss of 25%-50%, Grade IV represents loss of 50–75% of OPL and Grade V implies a loss from 75–100% of the layer.

### Quantification of retinal ganglion cells

Retinal ganglion cells (RGC) were counted manually in masked fashion in representative sagittal sections that contained the ONH. RGC were identified by shape and granularity in H/E staining. 10–12 visual fields were evaluated and numbers are given as RGC/mm.

### Electroretinography

Visual function was assessed using Ganzfeld electroretinography (ERG) according to standard methods as described in our previous works [16]. In short, animals were dark adapted overnight prior to measurement. Mice with the pupils dilated by 0.5% tropicamide and 2.5% phenylephrine hydrochloride were placed in a Ganzfeld bowl (Roland Consult, Brandenburg, Germany). Monopolar contact lens electrodes were applied as recording electrodes, and subcutaneously fixed platin needles served as reference electrodes. Scotopic ERG was recorded in the dark-adapted state. ERG was used to classify the degree of alteration comparing the BCCAO animals to sham. Animals with statistically significant difference at week 2 after BCCAO, were considered as “early affected” whereas the rest was kept as “late affected”.

### Statistical analysis

All experiments were repeated, at least, three times. The independent unit of observation (N) was each individual eye, although experimental groups and biometric N were based on animals. For all cases, unless indicated the contrary, values are expressed as mean ± SEM. S3 File compiles all numeric results presented in this work in any format. Statistical significance was calculated using t-test (paired when time-course analysis and unpaired when independent groups compared at a time-point) or ANOVA when comparing multiple groups. Mann-Whitney U test was applied to determine normal distribution of the data. P value is indicated in the figures when considered significant and expressed as * when p<0.05, ** when p<0.01 and *** when p<0.001.

### Bias assessment and bias prevention

Animals were randomized to receive micro-coil implantation (BCCAO group) or not (sham group). Sample size was determined according to previous publications using this model. Experiments were performed always at the same time and using the same facilities and equipment for in vivo analysis. Experiments were blind, and analysis was performed blind and often by other than the investigator that performed the experiment. Some animals died shortly after surgery or during the time-course of the experiment and were excluded from the study (N = 10 animals). We also established exclusion criteria depending on the read-out (Table 4).

**Table 4:** Exclusion criteria to reduce bias.

Read-out	Exclusion criteria
Scanning laser ophthalmoscope (SLO)	1. Cataract formation or opacity of the cornea/lens that does not allow reliable imaging after anesthesia. 2. Low quality of the image (e.g. blurriness, low edge sharpness).
Electroretinography (ERG)	1. No signal detected or delocalization of the electrodes. 2. Animals moved or died during ERG recording.
Immunohistochemistry	1. Tissue folds or excessive artefacts after histological processing.
Retinal whole-mounts	1. Loss of integrity of the tissue during retinal processing.
qPCR	1. Low quality of the RNA/DNA (260/280 ratio below 1.7).

## Results

### Retinal vein dilatation in BCCAO

**Fig 1:**
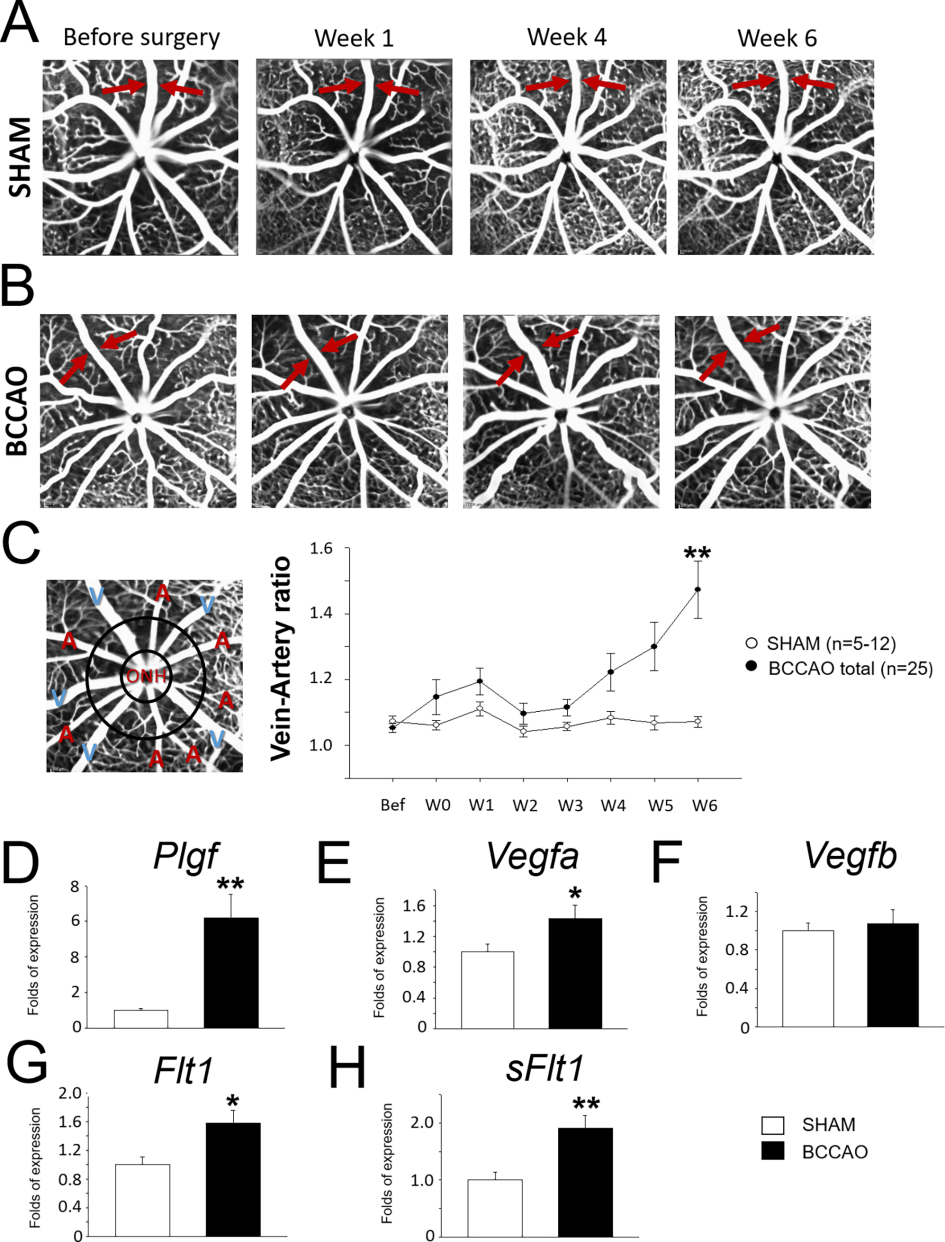
Vascular dynamics in the retina after BCCAO. **A.** Time-course of fundus angiographies after BCCAO (A) or sham intervention (B). Arrows point to (dilated) veins. **C.** Left: Representative fundus angiographies depicting arteries (A) and veins (V). Right: Bar chart represents the V-A ratio of the distinct groups (BCCAO vs. sham) during the time-course. **D-H.** Bar charts represent differential RNA expression of angiogenic markers in retinal tissue.

FAG was used to screen the vasculature *in vivo* weekly after micro-coil implantation (Fig 1A and 1B). Vascular dynamics were assessed distinguishing veins and arteries and calculating a relative ratio of the vessel width. Veins started to show dilatation after 3 weeks with BCCAO, but the vein-artery (V-A) ratio was significantly altered only 6 weeks after coil (Fig 1C). Among coiled animals there was a high variability in the degree of impact. A low percentage of BCCAO individual retinas (15%) did not show any vascular changes and behaved like sham controls. Furthermore, we classified as mild or severe vein dilatation in terms of absolute V-A ratio numbers, although the dynamics of the alteration were analogous (Fig 1C). *Ex vivo* data on retinal flat-mounts stained against Isolectin-B_4_ verified structural dilation of the veins (S2 Fig).

Using qPCR of retinal tissue, several markers associated with angiogenesis and vascular remodeling supported the previous findings. RNA levels of the ligand placenta growth factor (*Plgf*) were upregulated (Fig 1D), whereas vascular endothelial growth factor (*Vegf*)-*a* and -*b* only showed a trend of increase (Fig 1E and 1F). The receptors Fms-related Tyrosine Kinase 1 (*Flt1*) and its soluble form (*sFlt1*) RNA levels were significantly upregulated in the retina 6 weeks after BCCAO (Fig 1G and 1H).

### Time-course of distribution of mononuclear phagocytes in the retina

**Fig 2:**
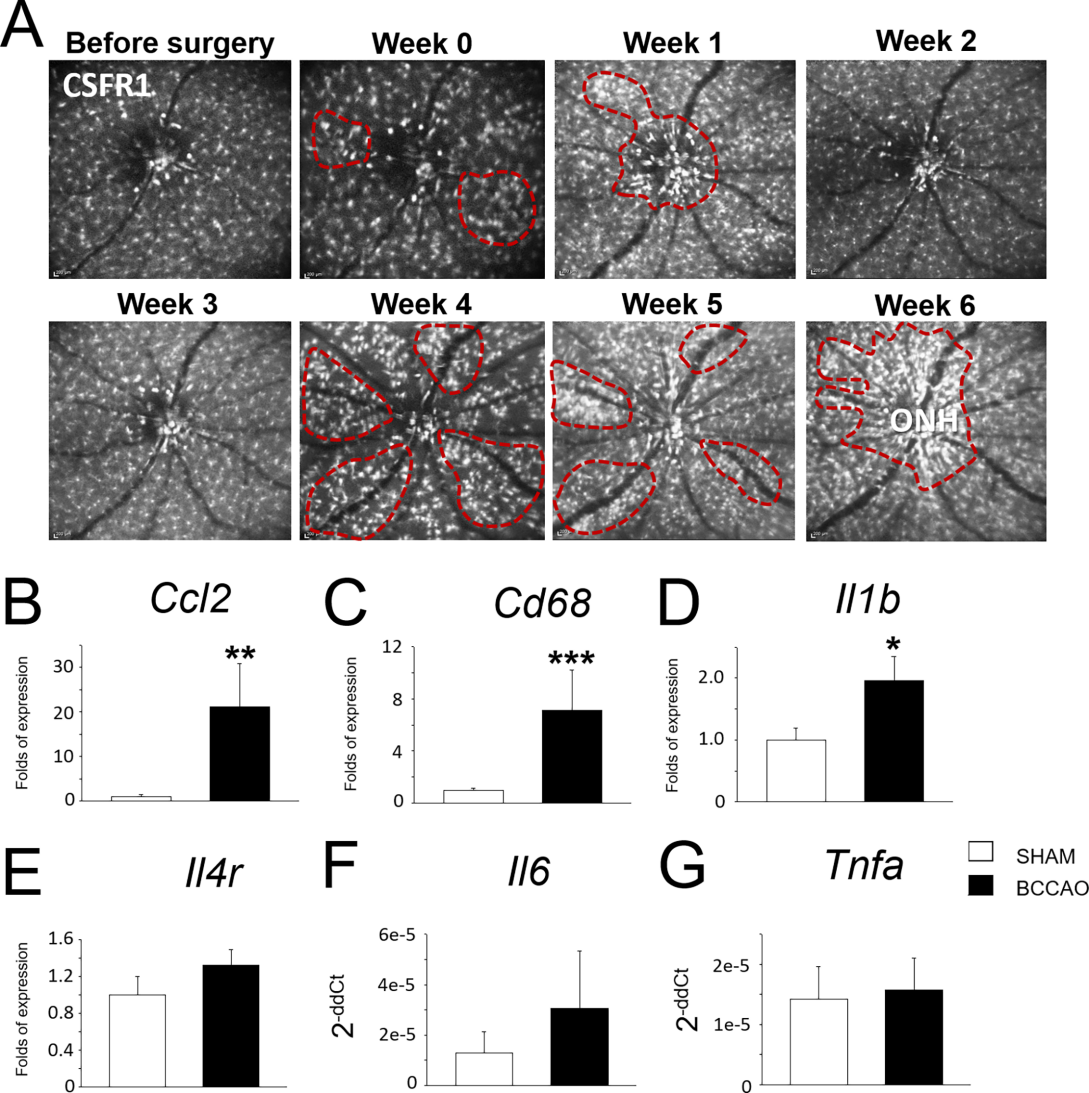
Inflammatory dynamics in the retina after BCCAO. **A.** Time-course of fundus auto-fluorescence of MacGreen mouse after BCCAO. EGFP signal corresponds to myeloid cells. Marked regions (dashed lines in red) show clusters of mononuclear phagocytes. **B.** Bar charts represent differential RNA expression of inflammatory markers of retinal tissue.

Inflammation was explored *in vivo* using MacGreen mice to monitor mononuclear phagocytes (MPs). Auto-fluorescence images were taken weekly and showed dynamic changes in the distribution of MPs as well in their activation state in the retina (Fig 2A, S1 Video). Remarkably, animals with micro-coil showed MP mobilization/accumulation surrounding veins (Fig 2A, dashed-lined area). The differences in the pattern of MP distribution, however, occurred at different time-points: some animals showed the dynamic changes rapidly at the same week after the coil implantation, whereas others did 3 weeks after (S1 Video). All animals with BCCAO showed alterations *in vivo* regarding MP turnover. MPs were accumulated surrounding the ONH area at the end-point of the experiment (Fig 2A, Week 6).

After BCCAO, RNA levels of several genes related to inflammation and MPs were upregulated in the retina: *Il6*, *Cd68* and *Ccl2*. *Il1b*, *Il4r* and *Tnfa*, however, did not show significant differences (Fig 2B).

### Differential temporary changes in visual function and grades of alteration

**Fig 3:**
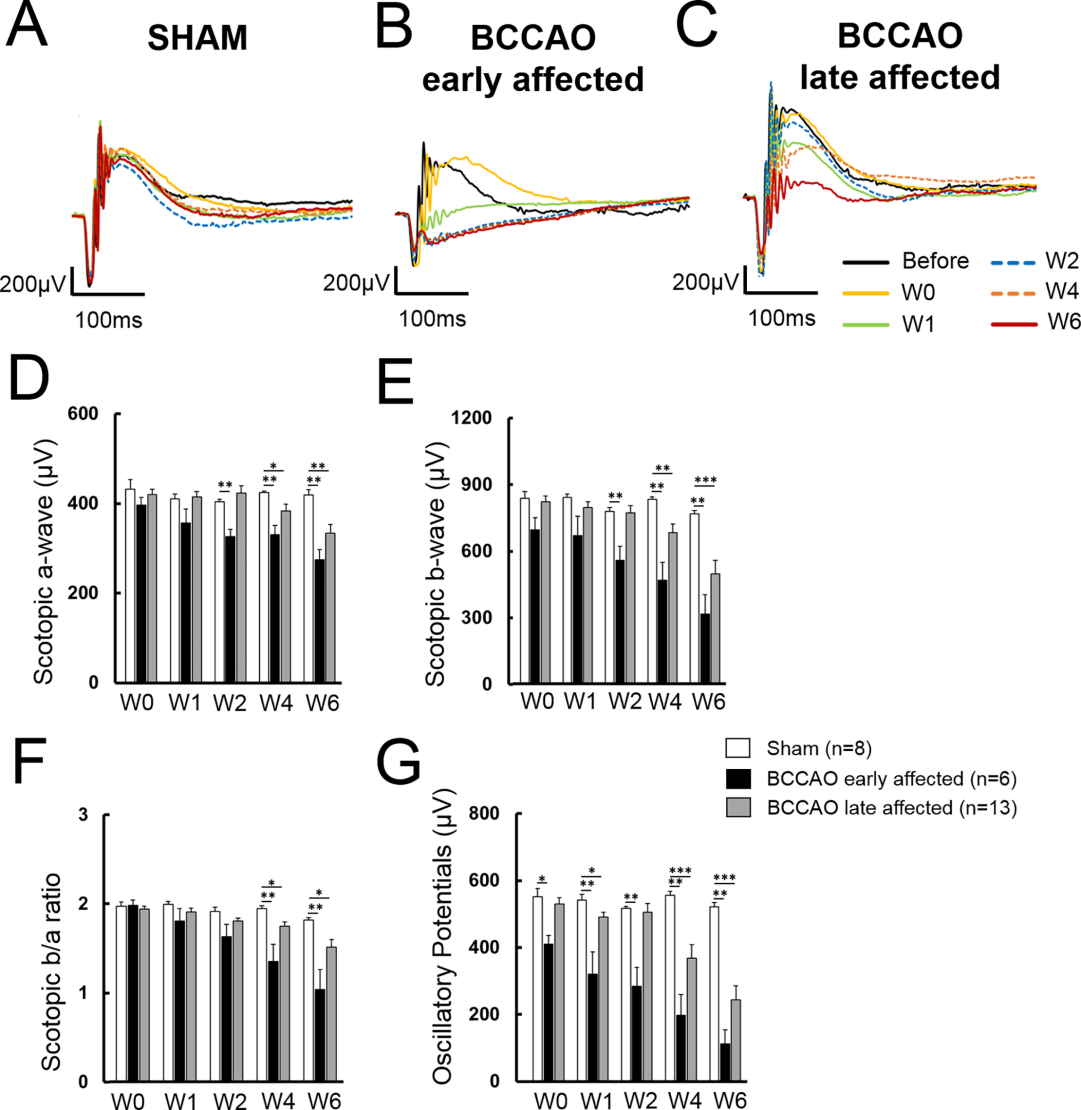
Analysis of temporal changes in visual function using Ganzfeld ERG. **A-C.** Graphs show representative curves of Ganzfeld ERG during the experimental time-course of a (A) sham, (B) late affected BCCAO and (C) early affected BCCAO animals. **D.** Scotopic a-wave. **E.** Scotopic b-wave. **F.** b/a-wave ratio. **G.** Oscillatory potentials.

Ganzfeld ERG was applied to assess retinal function. Under scotopic conditions, sham animals showed a physiological response curve with normal amplitudes for a-wave, b-wave and oscillatory potentials. No significant changes could be detected in control animals during the 6-week time-course after surgery (Fig 3A). In BCCAO conditions, however, the time course and the reduction of the different amplitudes of the curve was variable: some of the animals showed a mild reduction of the amplitudes at late stages after the micro-coil implantation (W4) and are referred as late affected (Fig 3B), whereas another group of animals showed an early and stable amplitude decrease in the first week after the surgery (W1) and we classified them as early affected (Fig 3C).

Altogether, a decrease in a-wave (Fig 3D), b-wave (Fig 3E) amplitudes was observed between sham and BCCAO animals, becoming significant at W2 for the early affected and at W4 for the late affected, respectively. B-a-ratio was significantly reduced at W4 in both BCCAO groups compared to sham (Fig 3F). Early affected animals showed significantly smaller amplitudes of oscillatory potentials already at W1, whereas late affected showed that feature one week after (Fig 3G). The decrease of oscillatory potentials is an indirect evidence for hypoxia in the retina. Protein expression of hypoxia-inducible factor 1-alpha (HIF-1α) in the retina of animals after 1 week (W1) with BCCAO further supported this finding (S3 Fig).

### Structural changes of the retinal layers

Retinal structure was evaluated systematically 1 and 6 weeks after BCCAO: 1) *ex vivo* in H/E sagittal sections and 2) *in vivo* with OCT retinal thickness mapping.

**Fig 4:**
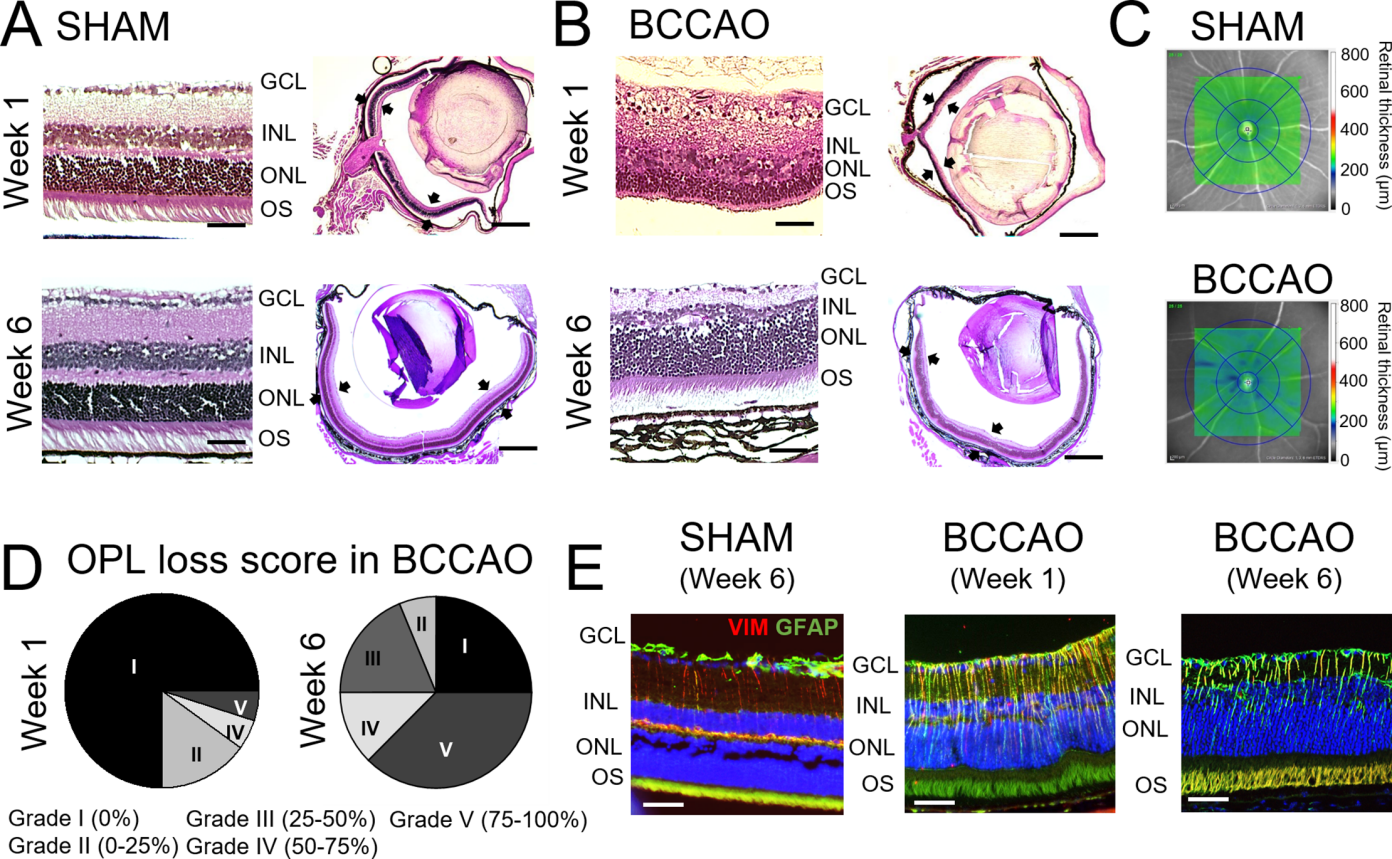
Structural changes in the retina after BCCAO. **A.** Representative sagittal sections of the eye stained with H/E showing the retinal layers in a control eye. **B**. Representative sagittal sections of the eye stained with H/E showing structural alterations in the retinal layers 1 and 6 weeks after BCCAO. Scale bar on magnification = 100 μm; Scale bar on full retina = 500 μm. Black arrows indicate the retina. **C.** OCT retinal mapping of representative sham and BCCAO animals. Colorimetric scale indicates retinal thickness. **D**. Pie charts show distribution of the animals 1 or 6 weeks after BCCAO according to loss of the outer retinal layer (OPL). Grading is indicated in the figure according to percentages in OPL loss. **E.** Representative sagittal sections of the retina co-stained against GFAP (green) and VIM (red) showing Müller cells. Nuclei were counterstained with DAPI (blue). Scale bar = 50 μm.

In H/E stained sections, a loss of the OPL was observed. The extent of loss of OPL varied among the animals with BCCAO, and sometimes even the adjacent nuclear layers such as the inner nuclear layer (INL) were affected (Fig 4A and 4B). We did not observe significant differences in numbers of RGC in coil compared to sham. BCCAO (N = 6 animals) presented 90 RGC/mm whereas sham (N = 3 animals) had 94 RGC/mm (p = 0.833). Using OCT retinal thickness mapping multiple retinal regions were identified indicating a significant thinning of the retina in coil after 6 weeks of BCCAO (Fig 4C, blue areas and S4 Fig). Sham did not show relevant variations in thickness (Fig 4C and S4 Fig).

The degree of loss of OPL was different among the animals that had coil implantation. Retinas were evaluated *ex vivo* and graded according to the percentage of OPL loss (Fig 4D). 1 week after coil implantation, only 10% of individual retinas showed structural changes of the OPL (Fig 4D, Week 1), although at week 6 most of the animals varied to a bigger or smaller extent (Fig 4D, Week 6). 25% of evaluated retinas with BCCAO did not show any loss of OPL.

Müller cells were studied as one of the main cell types contributing to retinal structure integrity. Using glial fibrillary acidic protein (GFAP), however, remarkable gliosis of the retina was observed compared to sham controls (Fig 4E).

### Synaptic changes in the neural retina

Synaptic proteins were evaluated in order to assess if they were affected by the loss of OPL at different time-points after coil implementation.

**Fig 5:**
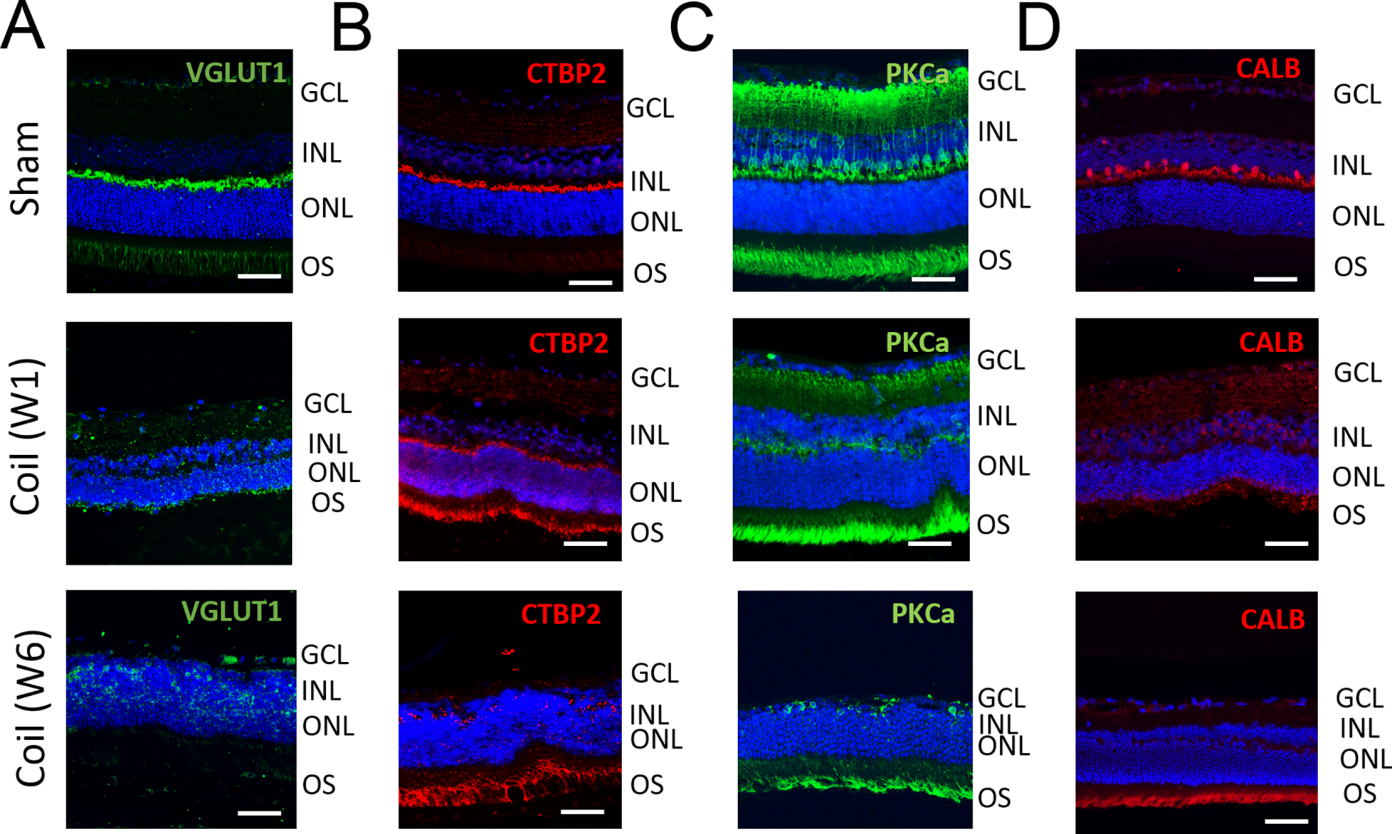
Delocalization of synaptic markers in the retina after BCCAO. **A.** Representative sagittal sections of the retina stained against VGLUT1 (green) showing loss and delocalization of pre-synaptic proteins in animals with BCCAO. **B.** Representative sagittal sections of the retina stained against CTBP2 (red) showing loss and delocalization of pre-synaptic proteins. **C.** Representative sagittal sections of the retina stained against PKCα (green) showing loss and delocalization of post-synaptic proteins after prolonged BCCAO. **D.** Representative sagittal sections of the retina stained against CALB (red) showing loss horizontal cells in coiled animals. All nuclei were counterstained with DAPI (blue). For all cases, scale bar = 50.

Retinal sagittal sections were stained with antibodies against vesicular glutamate transporter 1 (VGLUT1). VGLUT1 is associated with the membranes of synaptic vesicles and was used to detect the pre-synaptic site of the photoreceptor bipolar synapse. 1 week after BCCAO, VGLUT1 expression was disorganized compared to that in controls. This delocalization persisted 6 weeks after (Fig 5A).

C-terminal-binding protein 2 (CTBP2), which is a component of synaptic ribbons, served as another marker for the pre-synaptic site. Coil animals revealed delocalization and loss of CTBP2 expression at W6, whereas at W1 the staining pattern was still comparable to sham animals that presented an intense signal along the presynaptic site of the OPL (Fig 5B).

Bipolar cells were detected using an antibody against Protein kinase C α (PKCα). PKCα staining helps to identify the post-synaptic site of the synapse of photoreceptors and bipolar cells. PKCα staining revealed dislocalization and loss of arborization of the bipolar cells after BCCAO already after 1 week. (Fig 5C).

Horizontal cell loss was detected in animals with micro-coil one week after BCCAO using Calbindin-D28k (CALB) staining (Fig 5D).

## Discussion

Several diseases lead to visual impairment through ischemia. BCCAO is an established model for ischemic retinopathy that reproduces neurodegenerative features after blood flow reduction [4,17]. Most previous studies employed Wistar rats with complete ligation of the common carotid artery whereas we used a micro-coil in mice. This leads only to minor reduction in blood flow in the brain by approx. 20% [6]. Furthermore, previous studies focused mainly on the analysis of structural retinal alterations (e.g. thinning of the retina after 1 week to 2 months of artery ligation) [8,10,18,19]. Although in the literature RGC loss has been described as early as week 1 after BCCAO [8,18], we did not detect loss of RGC during the time course. Existent literature is only in rat models, but we believe that the extent of vessel occlusion, and thus hypoperfusion, might explain these discrepancies. In our model, we were also able to reproduce most of the findings regarding Müller cell gliosis, loss of horizontal cells and loss of synaptic protein [10,20,21]. Previous studies already described alterations in the OPL after BCCAO [8,10,20,21]. We provide additional structural details by determining delocalization or loss of OPL in terms of pre- and post-synaptic proteins. These findings were supported functionally by means of ERG. Here we detected a strong reduction in the b-wave, representing bipolar cell activity, in relation to the a-wave that represents the photoreceptor activity and was only minor affected in BCCAO. On the functional level, these data indicate a reduction in the signal transfer from the photoreceptors to the second neuron, the bipolar cells that correlates with a loss of photoreceptor synapses. Most works focus on the impact of BCCAO on retinal neurons, but there is not much data on vascular remodeling and inflammation concomitant to ischemia in this model. We showed dynamics of vein dilation in the time-course after BCCAO *in vivo*. Differential gene expression of angiogenic markers in affected animals furthermore supported these findings. Since BCCAO is primarily a vascular dementia model, the brain is clearly affected by the intervention. Both cerebral blood flow as well as behavioral and structural alterations are detectable in mice after BCCAO [6]. The loss of synapses that we detected in the neural retina, however, has not been assessed in brain sections of different areas yet.

Although it is of common sense that ischemia and inflammation are two related pathological processes, we have been the first to monitor the distribution and activation of MPs *in vivo*. Additionally, we provide evidences of differential gene expression regarding macrophage recruitment.

So far, animal models for retinal vascular and neurodegenerative disease were studied by analyzing only selected endpoints during the progression of the pathology [22–24]. Furthermore, for many of the cases these analyses have been done *ex vivo*.

SLO, OCT or ERG are *in vivo* non-invasive methods, affordable for most laboratories, and can be easily combined with transgenic models. Data acquisition *in vivo* has the advantage to enable follow-up studies using the same subject and thus minimizing the number of experimental animals.

The traditional approach with few fixed time-points defined by previous knowledge of time courses, nonetheless, might be useful for models with a well-characterized and established disease phenotype in a rather short lapse. One illustrative example is the laser-induced choroidal neovascularization [25], which is an acute model for wet age-related macular degeneration. However, most models that focus more on chronic aspects of diseases cannot be determined in such a short time scale and rigid manner. With our approach to analyze this chronic model weekly, we demonstrated that just few time-points do not deliver a full picture of the dynamic changes of the retina that might be critical for the understanding of the progression of the disease and for identification of critical points of intervention. Previous works in BCCAO only presented a small insight in the *in vivo* strategies to analyze the retina. Some groups explored the BCCAO model with total occlusion using ERG, but only acutely (14 days after occlusion). Their results are in accordance with our data [5,26,27]. Huang and colleagues analyzed the retina by means of FAG after 3 weeks of BCCAO in rats, and observed vein dilatation, increasing tortuosity and blurring of the optic disc, but only qualitatively [7].

Based on our data, we detected general patterns with differences in terms of severity and onset of the pathological changes after BCCAO. Albeit this variability hampers the statistical analysis, we detected significant differences even in composite analysis, revealing a high degree of robustness of the data. Findings presented important temporal differences between individuals undergoing the same treatment and, interestingly, even intra-individually among contralateral eyes. Thus, variability is a key parameter to determine the degree of alteration and to understand the effect of hypoperfusion of the retina in a model such as BCCAO.

For instance, vein dilatation does not follow a linear progression: there are hints of dilatation early after micro-coil implantation in some individuals classified as “severe”, as Huang and colleagues described [7], although later it fluctuates. Other less affected animals, showed same features with a lower degree of severity, and one-third group did not show pathological changes at all. Using auto-fluorescence measurements in MacGreen mice, we also showed how the distribution and activation of MPs in the retina is highly variable during the time-course of BCCAO among the individuals: after implantation of the micro-coil, only some mice showed MPs mobilization and clustering and, 2 weeks after, they switched to a quiescent state. At week 4, a second phase of activation occurred reaching a high degree of accumulation in the ONH area at week 6. This inflammatory phenomenon appeared with a delay of weeks in other animals. Oscillatory potentials diminished one week after BCCAO and hint to the ischemic state of the retina, but only after 4 weeks, we found a decrease in the a- and b-waves, suggesting damage in the photoreceptors and bipolar cells respectively. Immunohistochemistry data verified these findings and these are in accordance with other works published as argued above. It is interesting to note that photoreceptors appeared more affected compared to the neurons of the inner retina. This could be an effect of a differential reduction in the blood flow between the choroidal and the retinal circulation. However, no specific data exist that describe the proportion of blood flow between the retinal and choroidal perfusion. Moreover, it seems that there are intersections between the optic nerve, retinal and choroidal blood supply with strong individual variations [28]. Given the estimation of blood flow reduction in the central nervous system by 15%, we argue the same effects for both retinal and choroidal circulation. Thus, we are not able to deliver a concluding explanation for the primal affection of the photoreceptor synapse by a differential blood flow reduction between retinal and choroidal circulation.

The above described inter- and intra-individual variability in both, onset and severity of alteration, demonstrates that factors beyond genetics can have major impact on disease phenotype of this model. As these animals are inbred, and we used highly standardized experimental approaches performed by well-trained surgeons and experimentalists, minor variations in experimental conditions, housing, handling, etc. seem to affect our model. While such experimental variability may seem as a nuisance, it probably reflects the clinical condition in of inter- and intra-individual disease progression and response to therapy.

*In vivo* monitoring over the entire time span of an experiment not only reveals such variability, but may also help to understand individual disease progression and therefore improve bench to bedside translation.

BCCAO is a model that has not been only used for investigation of hypoxic impact onto the retina but also onto the brain. The BCCAO impacts onto the retina point to another confounding effect when using this model in behavioral tests for brain research. Our data lead us to the conclusion that BCCAO leads to blindness at variant time points from blindness of very early onset to mild effects after 6 weeks. The blindness develops by an interruption of synaptic connection between photoreceptors and bipolar cells. Blindness might confound behavioral testing of higher brain functions. Thus, in BBCAO ERG recording should be correlated with behavioral tests.

BCCAO calls for caution in drawing conclusions: limited experimental read-outs might lead to interpretation bias. Especially, translation medicine should consider individual time-courses and subgroups to approach therapeutic or interventional studies. Personalized medicine is something demanded in the clinic but, often, forgotten in basic research.

Our data have implications not only for the BCCAO model but also for the use of the new upcoming techniques in *in vivo* analysis models in basic translational research. In BCCAO, fixed time points for analysis might lead to wrong or contradictory conclusions. For the use of the variety of new *in vivo* analysis technologies, we demand to exploit fully their advantages and opportunities. These are multimodal analysis, close correlation with clinical assessments and follow-up studies; the latter one also reduces the number of animals in a study.

## Supporting information

S1 Fig Immunohistochemistry isotype controls. **A-B**. Sagittal sections of the retina stained against the secondary antibodies indicated in Table 3. Staining served as negative controls. Nuclei were counterstained with DAPI (blue). Scale bar = 50 μm.
(TIF)

S2 Fig Vein-artery ratio supplement. **A**. Representative retinal flat-mount stained against isolectin-B4. Representative artery “A” and vein “V” are indicated in the figure. Scale bar = 100 μm. **B.** Plot with the differences in vein-artery ratio. ** indicates p<0.01.
(TIF)

S3 Fig HIF1α in the retina. **A-C**. Representative sagittal sections of the retina stained against HIF1α (red) in (**A**) sham and BCCAO (**B**) 1 week and (**C**) 6 weeks after implantation. Nuclei were counterstained with DAPI (blue). Scale bar = 50 μm.
(TIF)

S4 Fig Retinal thickness mapping supplement. **A.** Colorimetric grading of retinal thickness in a sham and BCCAO animals. Images were obtained using Eye Heidelberg software and are supplementary to the ones displayed in Fig 4C without infra-reflectance background. **B.** Representative OCT used by the software to create the retinal thickness maps in a sham and a BCCAO animal. The OCT image shows differences in layer thickness. Red line displayed in the images represents the area selected for thickness mapping.
(JPG)

S1 Video Dynamic time-course of fundus auto-fluorescence of representative MacGreen mouse sham and after BCCAO (early and late affected animals). EGFP signal (white) corresponds to myeloid cells. Red arrowheads point activated mononuclear phagocytes.
(MP4)

S1 File ARRIVE guidelines checkpoint list. Checklist of aspects related to animal research in in vivo experiments.
(PDF)

S2 File Plos one humane checklist endpoints. Checklist of aspects from the study design which include death of a regulated animal as a likely outcome or planned experimental endpoint.
(PDF)

S3 File Numeric result compilation. Results for each analysis carried out with a measure of precision (SEM).
(DOCX)
