# Correction: Individual and temporal variability of the retina after chronic bilateral common carotid artery occlusion (BCCAO)

**DOI:** 10.1371/journal.pone.0196538

**Published:** 2018-04-23

**Authors:** Sergio Crespo-Garcia, Nadine Reichhart, Sergej Skosyrski, Marco Foddis, Jim Wu, Aleksandar Figura, Christina Herrspiegel, Martina Füchtemeier, Celeste Sassi, Ulrich Dirnagl, Antonia M. Joussen, Olaf Strauss

The following information is missing from the Funding section: The authors acknowledge support from the German Research Foundation (DFG) and the Open Access Publication Fund of Charité –Universitätsmedizin Berlin.
