# Supplementary figures and images for: Individual and temporal variability of the retina after chronic bilateral common carotid artery occlusion (BCCAO)
Source: PLoS One. 2018 Mar 16;13(3):e0193961. doi: 10.1371/journal.pone.0193961 (PMC5856268; doi:10.1371/journal.pone.0193961)

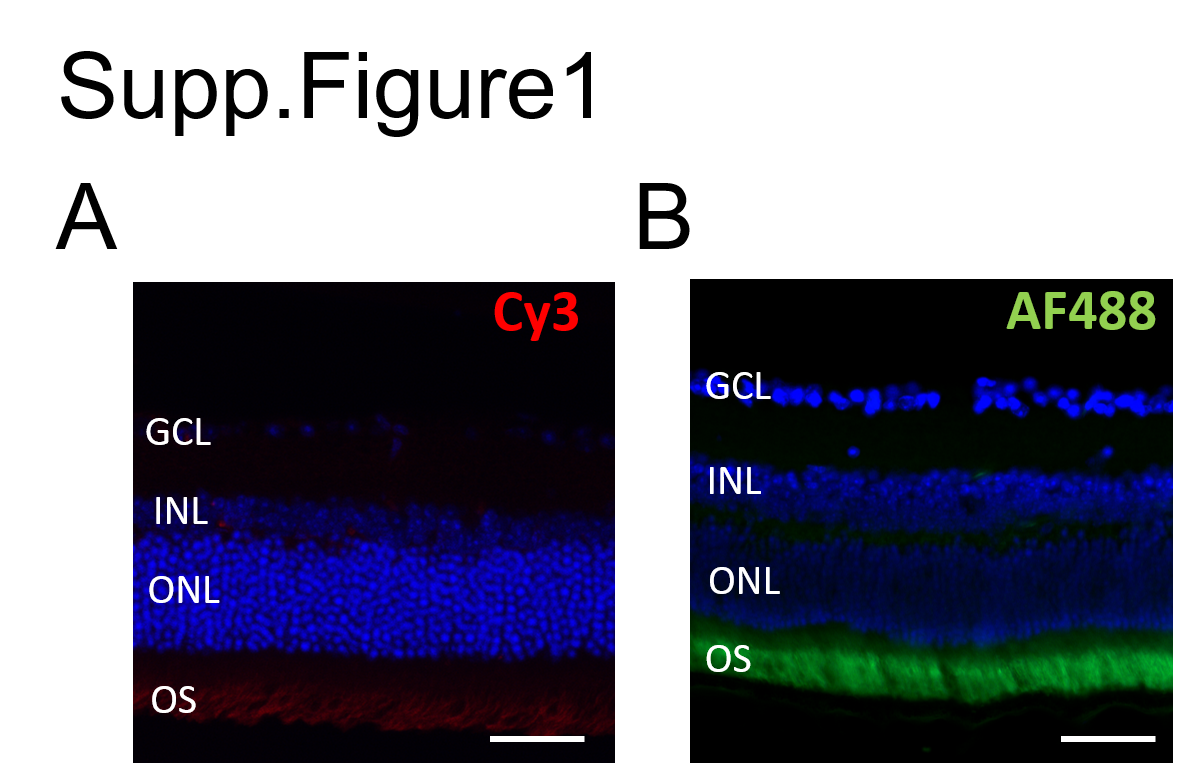

Supplement: S1 Fig — A-B. Sagittal sections of the retina stained against the secondary antibodies indicated in Table 3. Staining served as negative controls. Nuclei were counterstained with DAPI (blue). Scale bar = 50 μm. (TIF) [file pone.0193961.s001.tif]

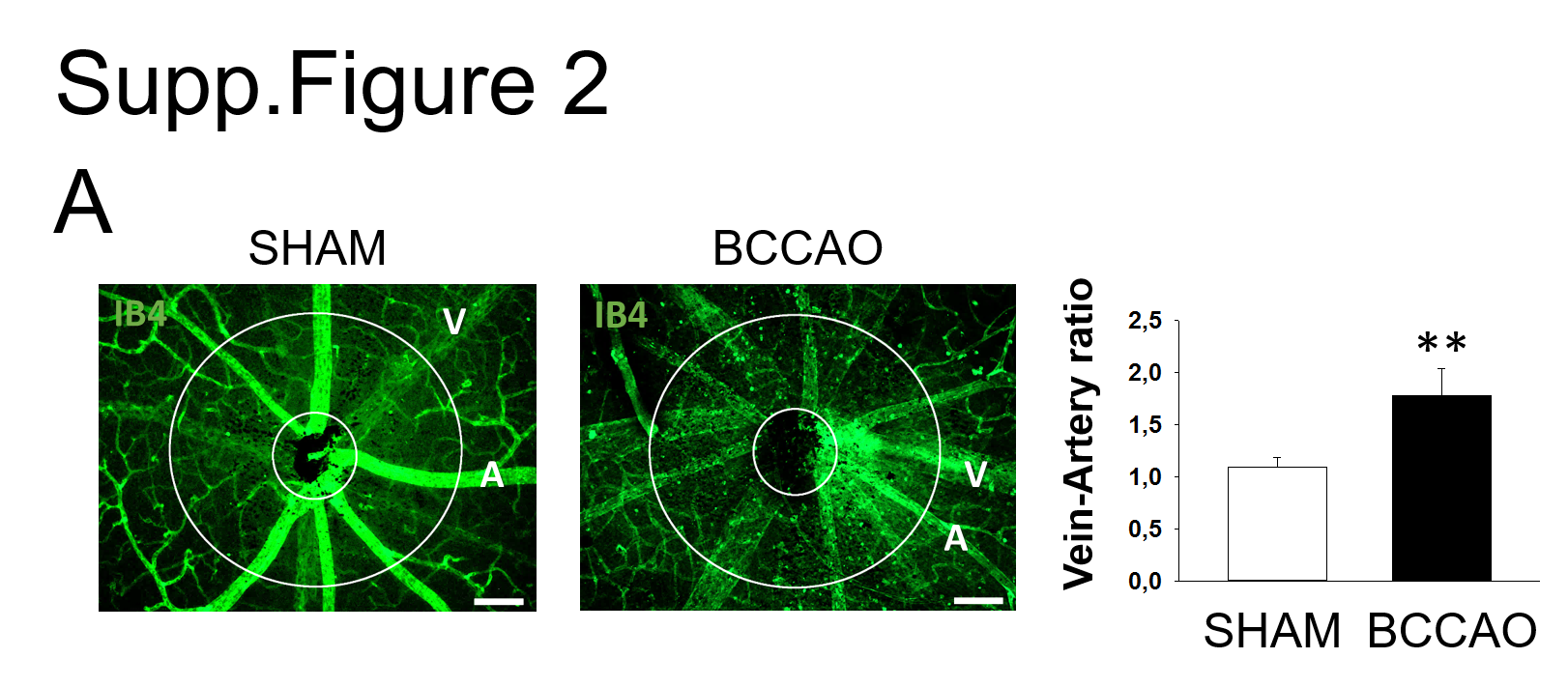

Supplement: S2 Fig — A. Representative retinal flat-mount stained against isolectin-B4. Representative artery “A” and vein “V” are indicated in the figure. Scale bar = 100 μm. B. Plot with the differences in vein-artery ratio. ** indicates p<0.01. (TIF) [file pone.0193961.s002.tif]

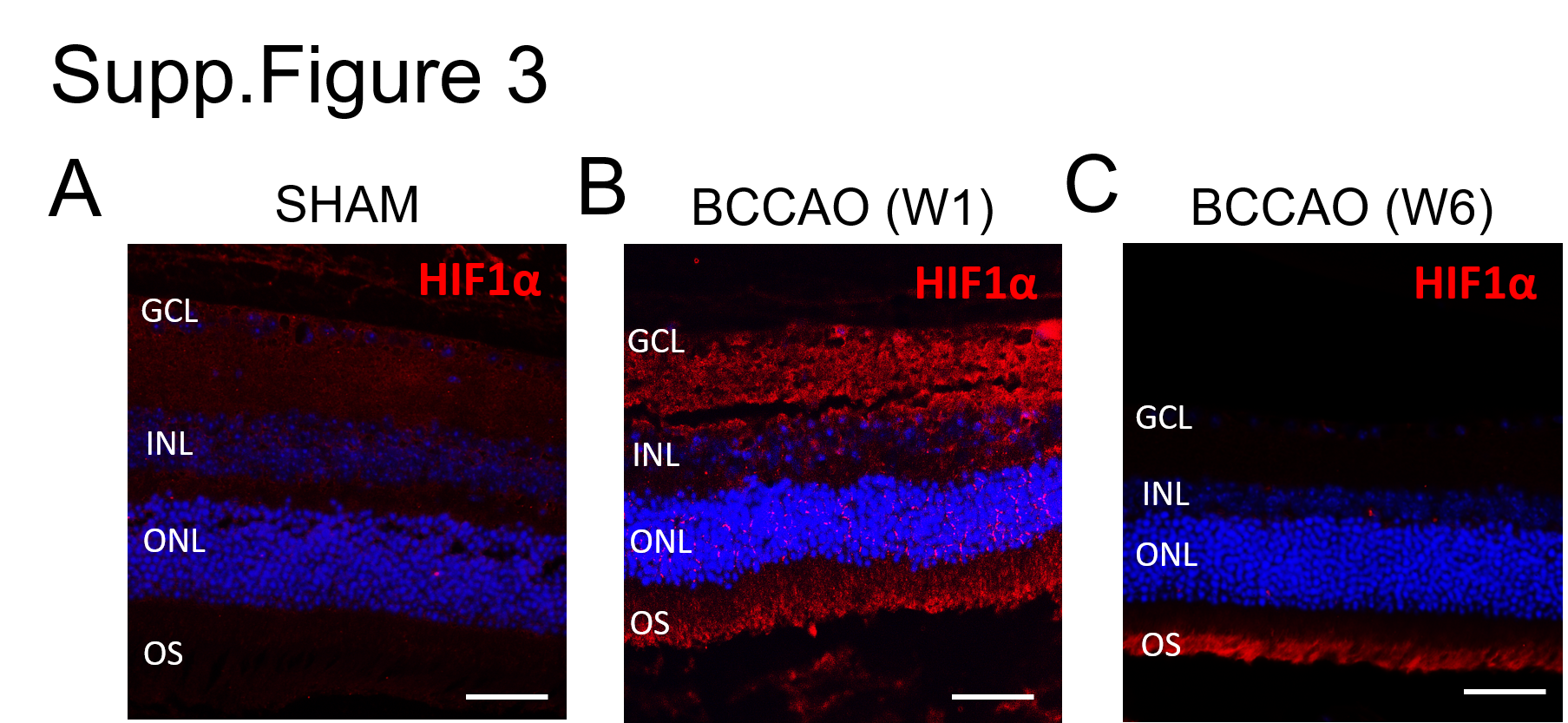

Supplement: S3 Fig — A-C. Representative sagittal sections of the retina stained against HIF1α (red) in (A) sham and BCCAO (B) 1 week and (C) 6 weeks after implantation. Nuclei were counterstained with DAPI (blue). Scale bar = 50 μm. (TIF) [file pone.0193961.s003.tif]

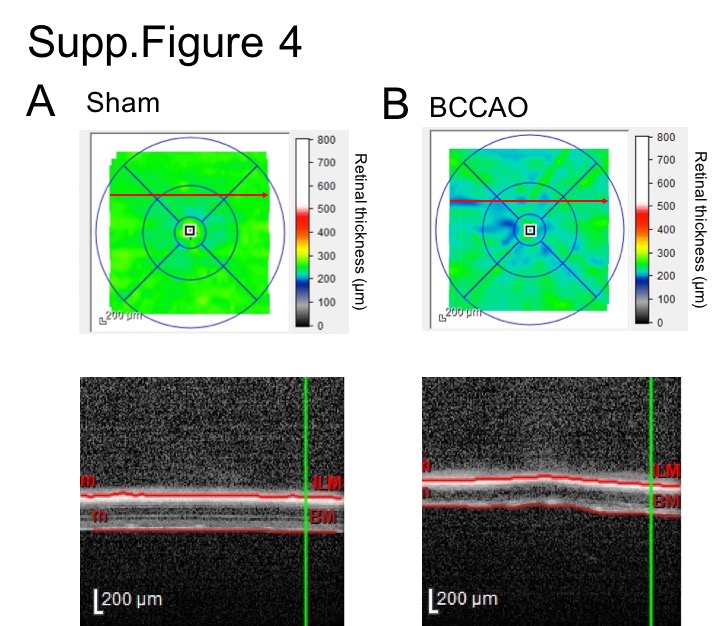

Supplement: S4 Fig — A. Colorimetric grading of retinal thickness in a sham and BCCAO animals. Images were obtained using Eye Heidelberg software and are supplementary to the ones displayed in Fig 4C without infra-reflectance background. B. Representative OCT used by the software to create the retinal thickness maps in a sham and a BCCAO animal. The OCT image shows differences in layer thickness. Red line displayed in the images represents the area selected for thickness mapping. (JPG) [file pone.0193961.s004.jpg]
