# Supplementary material for: Individual and temporal variability of the retina after chronic bilateral common carotid artery occlusion (BCCAO)
Source: PLoS One. 2018 Mar 16;13(3):e0193961. doi: 10.1371/journal.pone.0193961 (PMC5856268; doi:10.1371/journal.pone.0193961)
Supplement: S3 File — Results for each analysis carried out with a measure of precision (SEM). (DOCX) [file pone.0193961.s008.docx]

**Supporting information**

**S1 Supporting data.** Results for each analysis carried out with a measure of precision (SEM).

Vein-artery ratio from fundus angiography data corresponding to graph in Figure 1C:

|  | Sham | | BCCAO | |
| --- | --- | --- | --- | --- |
|  | mean | ±SEM | mean | ±SEM |
| **BEFORE** | 1,072 | 0,0171 | 1,0526 | 0,0139 |
| **WEEK0** | 1,0606 | 0,0148 | 1,1463 | 0,0539 |
| **WEEK1** | 1,1109 | 0,0206 | 1,1939 | 0,041 |
| **WEEK2** | 1,0418 | 0,0162 | 1,0964 | 0,0319 |
| **WEEK3** | 1,0572 | 0,0127 | 1,1148 | 0,0251 |
| **WEEK4** | 1,0837 | 0,019 | 1,2221 | 0,0571 |
| **WEEK5** | 1,0679 | 0,0207 | 1,2998 | 0,0735 |
| **WEEK6** | 1,0713 | 0,0163 | 1,4724 | 0,0865 |

Vein-artery ratio data from flatmount staining corresponding to graph in supplementary information Supp. Figure 2:

|  | Sham | | BCCAO | |
| --- | --- | --- | --- | --- |
|  | mean | ±SEM | mean | ±SEM |
| **WEEK** 6 | 1,0925 | 0,0914 | 1,775 | 0,2606 |

Quantitative PCR data corresponding to angiogenic markers in Figure 1D-H:

| Gene | ***Vegfa*** | | ***Plgf*** | | ***Flt1*** | | ***sFlt1*** | | ***Vegfb*** | |
| --- | --- | --- | --- | --- | --- | --- | --- | --- | --- | --- |
|  | Sham | BCCAO | Sham | BCCAO | Sham | BCCAO | Sham | BCCAO | Sham | BCCAO |
| mean | 1,000 | 1,434 | 1,000 | 6,197 | 1,000 | 1,579 | 1,000 | 1,911 | 1,000 | 0,084 |
| ±SEM | 0,097 | 0,174 | 0,116 | 1,307 | 0,111 | 0,183 | 0,146 | 0,225 | 1,073 | 0,146 |

Quantitative PCR data corresponding to angiogenic markers in Figure 2B-G:

| Gene | ***Il1b*** | | ***Cd68*** | | ***Il4r*** | | ***Ccl2*** | |
| --- | --- | --- | --- | --- | --- | --- | --- | --- |
|  | Sham | BCCAO | Sham | BCCAO | Sham | BCCAO | Sham | BCCAO |
| mean | 1 | 1,962 | 1 | 7,141 | 1 | 0,1943 | 1 | 21,14 |
| ±SEM | 0,1894 | 0,39 | 0,1319 | 3,072 | 1,317 | 0,1722 | 0,4049 | 9,712 |

Electroretinography data corresponding to bar charts in Figure 3D-G:

|  |  | | Week 0 | | Week 1 | | Week 2 | | Week 4 | | Week 6 | |
| --- | --- | --- | --- | --- | --- | --- | --- | --- | --- | --- | --- | --- |
|  |  |  | Sham | BCCAO | Sham | BCCAO | Sham | BCCAO | Sham | BCCAO | Sham | BCCAO |
| **b/a ratio** |  | mean | 1,9 | 1,8 | 2,01 | 1,94 | 1,93 | 1,75 | 1,97 | 1,56 | 1,83 | 1,30 |
|  |  | ±SEM | 0,0 | 0,1 | 0,03 | 0,07 | 0,05 | 0,07 | 0,03 | 0,11 | 0,03 | 0,14 |
|  |  |  |  |  |  |  |  |  |  |  |  |  |
| **b-wave** |  | mean | 848,6 | 756,5 | 842 | 738,4 | 778 | 658,1 | 833 | 556,4 | 768 | 432,2 |
|  |  | ±SEM | 25,2 | 51,9 | 15,4 | 46,5 | 17,7 | 40,1 | 11,0 | 53,2 | 14,2 | 67,1 |
|  |  |  |  |  |  |  |  |  |  |  |  |  |
| **a-wave** |  | mean | 450,8 | 435,0 | 410 | 388,4 | 404 | 380,2 | 424 | 351,6 | 419 | 306,0 |
|  |  | ±SEM | 16,4 | 12,2 | 10,9 | 18,5 | 5,3 | 15,0 | 3,8 | 15,9 | 11,1 | 21,9 |
|  |  |  |  |  |  |  |  |  |  |  |  |  |
| **Oscillatory potentials** |  | mean | 536,6 | 424,2 | 542 | 413,0 | 517 | 394,8 | 557 | 285,9 | 521 | 182,9 |
|  |  | ±SEM | 21,1 | 38,0 | 16,6 | 37,0 | 6,3 | 37,0 | 11,6 | 47,2 | 14,2 | 42,4 |
